# Distribution of Shrubland and Grassland Soil Erodibility on the Loess Plateau

**DOI:** 10.3390/ijerph15061193

**Published:** 2018-06-07

**Authors:** Xiao Zhang, Wenwu Zhao, Lixin Wang, Yuanxin Liu, Qiang Feng, Xuening Fang, Yue Liu

**Affiliations:** 1 State Key Laboratory of Earth Surface Processes and Resource Ecology, Faculty of Geographical Science, Beijing Normal University, Beijing 100875, China; zhangxiao9005@gmail.com (X.Z.); yuanxinliu@rcees.ac.cn (Y.L.); 201231190029@mail.bnu.edu.cn (Q.F.); summerfxn@126.com (X.F.); 201531190025@mail.bnu.edu.cn (Y.L.); 2 Institute of Land Surface System and Sustainable Development, Faculty of Geographical Science, Beijing Normal University, Beijing 100875, China; 3 Department of Earth Sciences, Indiana University–Purdue University Indianapolis (IUPUI), Indianapolis, IN 46202, USA; lxwang@iupui.edu; 4 State Key Laboratory of Urban and Regional Ecology, Research Center for Eco-Environmental Sciences, Chinese Academy of Sciences, Beijing 100085, China; 5 College of Forestry, Shanxi Agricultural University, Taigu, Shanxi 030801, China; 6 Center for Human-Environment System Sustainability, Beijing Normal University, Beijing 100875, China

**Keywords:** soil erodibility, models, shrubland, grassland, Loess Plateau

## Abstract

Soil erosion is one of the most severe problems facing environments and has increased throughout the 20th century. Soil erodibility (*K*-factor) is one of the important indicators of land degradation, and many models have been used to estimate *K* values. Although soil erodibility has been estimated, the comparison of different models and their usage at a regional scale and, in particular, for different land use types, need more research. Four of the most widely distributed land use types were selected to analyze, including introduced and natural grassland, as well as introduced and natural shrubland. Soil particle size, soil organic matter and other relevant soil properties were measured to estimate soil erodibility in the Loess Plateau. The results show that: (1) the erosion productivity impact calculator (EPIC) model and SHIRAZI model are both suitable for the Loess Plateau, while the SHIRAZI model has the advantage of fewer parameters; (2) introduced grassland has better ability to protect both the 0–5 cm soils and 5–20 cm soils, while the differences between introduced and natural shrubland are not obvious at a catchment scale; (3) the *K* values of introduced grassland, natural grassland, introduced shrubland and natural shrubland in the 0–5 cm layer vary from 0.008 to 0.037, 0.031 to 0.046, 0.012 to 0.041 and 0.008 to 0.045 (t·hm^2^·h/(MJ·mm·hm^2^)), while the values vary from 0.009 to 0.039, 0.032 to 0.046, 0.012 to 0.042 and 0.008 to 0.048 (t·hm^2^·h/(MJ·mm·hm^2^)) in the 5–20 cm layer. The areas with a mean multiyear precipitation of 370–440 mm are the most important places for vegetation restoration construction management at a regional scale. A comprehensive balance between water conservation and soil conservation is needed and important when selecting the species used to vegetation restoration. This study provides suggestions for ecological restoration and provides a case study for the estimate of soil erodibility in arid and semiarid areas.

## 1. Introduction

Soil erosion is one of the most severe problems related to the environment, society and the economy [1,2]. Soil erosion has increased throughout the 20th century [3,4]. It not only causes land degradation, but also threatens local food security and social sustainability [1]. Soil erosion may contain multiple erosion types, including wind erosion, water erosion, gravity erosion and freeze-thaw erosion [5,6]. Scientists have realized that, for the control of soil erosion, it is necessary to provide policy makers and soil scientists with information about the processes of soil erosion based on a reliable assessment of vulnerability and risk levels [7]. Soil erodibility is the ease with which soil is detached by splash during rainfall, by surface flow or both [8], and it is one of the important indicators of land degradation [9,10,11]. Existing studies at the arid and semiarid places in Anatolia [12], Argentina [13], Australia [14], China [15,16,17], Morocco [18], Iran [19,20,21], Spain [22] and United states [23] have shown that soil erodibility is influenced by global climate change [24] and land use change [9,10,11,25]. The changes of land use types alter the vegetation coverage and then influence the soil quality and soil properties such as the soil particle distribution, soil organic carbon content and soil texture [19,23]. Runoff plots and check dams are the traditional monitoring methods [20,26,27]. Recently, the development of GIS has provided new methods for soil erosion research [28,29], such as geomatics approach [30] and geospatial approach [31], and electrical resistivity tomography [32] has also been used for in situ measurement. In the data-scarce regions, genetic algorithm can be utilized to estimate the average erodibility parameters [33]. These new methods make the mapping of soil erodibility faster and keep the land surface undisturbed, although their accuracy needs more verification, especially when predicting the soil erodibility in hilly areas.

China has more severe soil erosion than other countries, which has resulted in many problems [3,34]. The Loess Plateau that is located in the middle part of China has both the largest and deepest loess deposit in the world. The thickness of loess soil layers is usually 50–200 m, and some places may even reach more than 300 m depth [35]. This area has long been one of the most severely eroded areas on earth, especially in its loess hilly region [36]. The northern part of the Loess Plateau has been severely affected by wind–water erosion, and water erosion has gradually become the main type of erosion in the southern part of the Loess Plateau [37]. Many soil and water conservation engineering measures, such as terracing and check-dam constructions, have been used since the 1970s and achieve good results [36,38]. In addition to these engineering measures, the Chinese government implemented the *Grain to Green* project in 1999 to protect soil and water [39,40]. After more than 10 years, the coverage of vegetation has increased markedly in the Loess Plateau [41,42]. The Yellow River sediments reduced significantly, while the amount of river flows reduced as well [43]. One reason is the existence of plants, which protect the water from running away rapidly after rainfall, so more water can get into the soil; another reason is the growth of plants using a large amount of water [1,44,45]. So, for future vegetation restoration, species with lower water consumption rates and higher soil protection ability should be used to avoid further soil degradation [37,46].

The concept of soil erodibility is commonly represented by the *K* factor, which is defined as the average rate of soil loss per unit of rainfall erosion index from a cultivated continuous fallow plot, on a 9% slope 22.1 m long, by the universal soil loss equation (USLE) and revised USLE (RUSLE) [8,47,48]. *K*-factor values are best obtained from long-term direct measurements on natural runoff plots [49,50], but this method is hard to use at a large scale. So, the estimate of *K* values by using soil physical or chemical properties attracts more attention [50,51,52,53,54,55]. There is a long history of the research on soil erodibility, and USLE/RUSLE are the most widely used models in the world [56,57,58,59,60] to analyze the distribution of *K* or identify the erosion risk zones [61,62,63]. Many new models and indicators are emerging constantly to predicting the soil erodibility or evaluate the certainty of modeled data [64,65,66,67,68], such as Jet Erosion Device (Jed) [69,70,71], artificial neural network (ANN) model [72], erosion productivity impact calculator (EPIC) model [73] and rangeland hydrology and erosion model (RHEM) [74]. Most of them are based on the principle of USLE/RUSLE and have been used regionally [75]. There are also many models that have been used to estimate *K* values in the Loess Plateau, although the accuracy of results differs between different soil and land use conditions. For example, the *K* values estimated with NOMO model would considerably over-predict the rate of soil loss, so it is important to choose the model that is the most suitable for the research areas [44,49,76].

According to the existing studies, USLE/RUSLE model, NOMO model, EPIC model, SHIRAZI model and TORRI model are commonly used for estimating the *K* value in China [35,44,49]. For specific land use types, the comparison of different models and their usage at the regional scale need more research, although the *K* value of these models has been estimated. The Loess Plateau has a thick soil layer which is loose, and the surface soil is highly susceptible to erosion, so the study of subsurface erodibility is also important because it reflects the potential erosion of the soil [77,78,79]. This study chose the most widely distributed land use types (introduced grassland, natural grassland, introduced shrubland and natural shrubland) at catchment scale and regional scales [41,80,81], and estimated *K* values both in 0–5 cm (surface) soil layers and 5–20 cm (subsurface) soil layers. Other common land use types were also analyzed as a comparison at the catchment scale. This paper aims to: (1) compare and choose the most suitable models for *K* calculation at the small scale of the hilly areas in the Loess Plateau; (2) calculate *K* at catchment and regional scales by using the most suitable models and analyze the spatial distribution of soil erodibility at the regional scale to provide support to local soil conservation; (3) provides suggestions for the ecological restoration in arid and semiarid areas.

## 2. Materials and Methods

### 2.1. Description of the Sites

The runoff plots (109.32° E, 36.86° N) is located on 5°, 15° and 25° slopes at the south part of the Ansai catchment. They belong to Ansai Research Station of Soil and Water Conservation, Chinese Academy of Sciences. The horizontal projection area of each plot is 4 m × 10 m. The aspect of the slope is 82° east by north.

The Ansai catchment (108.01°–109.44° E, 36.51°–37.32° N) is located in the upper reaches of the Yanhe River. This area belongs to a typical loess hilly region and the mid-temperate continental semiarid monsoon climate region. The topography is fragmented and the soil erosion is heavy [82]. Grassland (24% to be included), shrubland (22% to be included), forest (24% to be included), farmland (15% to be included) and orchard (15% to be included) are the most widely distributed land use types in this catchment [81,83].

The regional scale research areas are along the north–south sampling belt, which is located in the north-central Loess Plateau of China. The sampling areas are Yijinghuoluo county (108.97°–110.42° E, 38.93°–39.82° N), Shenmu county (109.67°–110.90° E, 38.22°–39.45° N), Suide county (110.07°–110.68° E, 37.27°–37.75° N), Ansai county (108.10°–109.44° E, 36.51°–37.32° N), Baota district (109.24°–110.85° E, 36.18°–37.03° N) and Fuxian county (108.50°–109.72° E, 35.74°–36.39° N) from the north to the south. The mean annual precipitation calculated over multiyear periods ranges from 300 to 600 mm [81,84]. The precipitation in a given year is unevenly timed, and more than 60% falls during the growing season (from May to September) [85]. In the summer, the heavy rainfall usually results in soil and water losses [86]. Grasslands which are dominated by *Medicago sativa* or *Stipa bungeana* and shrublands which are dominated by *Hippophae rhamnoides* or *Caragana korshinskii* are the most widely distributed land use types at the regional scale [42,44,87].

### 2.2. Experimental Design

At the runoff plots, the grassland with introduced species (G1), grassland with natural species (G2), farmland (FA) and shrubland with natural species (S2) plots in each slope were chosen as the RUSLE estimate points. Precipitation was monitored by HOBO Rain Gauge Data Logger (RG3-M resolution: 0.2 mm; Onset Computer Corporation, Bourne, Massachusetts, USA). The vegetation coverage of each plot was measured by photographic method [88,89]. The soil loss for each plot was measured from June 2013 to September 2014. After each precipitation, the soil-water suspension in the runoff-collecting bucket was collected. Then, the suspension samples were allowed to sit for 24 h to make the sediment separate from the water, and the sediment was dried in an oven at 105 °C to a constant weight. The mean soil loss (*A* in RUSLE model) was calculated as the annual average value of the ratio of the sediment mass to the area of the plot [26,27,90,91]. The runoff-collecting buckets were cleaned after each collection of soil-water suspension samples.

At the Ansai catchment, the distribution of sampling points was as uniform as possible in order to make the sampling points more representative. With the help of Google Earth, the sampling areas were chosen along most of the main tributaries inside the catchment. The area of the chosen sampling places is more than 30 m × 30 m for each land use type and the sampling plots were set at the center of the sampling places. Sampling places of the same land use type were more than 100 m apart, and 133 sample points in total were chosen at this catchment. They included 11 introduced grassland points (G1), 25 natural grassland points (G2), 18 introduced shrubland points (S1), 15 natural shrubland points (S2), 37 forest points (FO), 23 farmland points (FA) and 23 orchard points (OR). The sampling points of G1, G2, FA and S2 were divided into three groups by the slope (less than 10°, 10–20° and more than 20°) to match the sample data obtained from the runoff plots (5°,15°,25°) when comparing the estimate methods results with the RUSLE estimate result.

At the regional scale, 62 sample points were chosen, which included 14 introduced grassland points (G1), 14 natural grassland points (G2), 20 introduced shrubland points (S1) and 13 natural shrubland points (S2). For each land use type, there were two to four sampling points in each sampling area at the south–north sample belt. The precipitation was collected at 62 Loess Plateau meteorological monitoring stations of China Meteorological Administration from 1998–2012. With the landscape type and administrative boundary in research area, the spatial distribution of precipitation was calculated by the weighted interpolating method (IDW) in ArcGIS 10.0, and the precipitation isoline interval was set to 70 cm.

**Figure 1:**
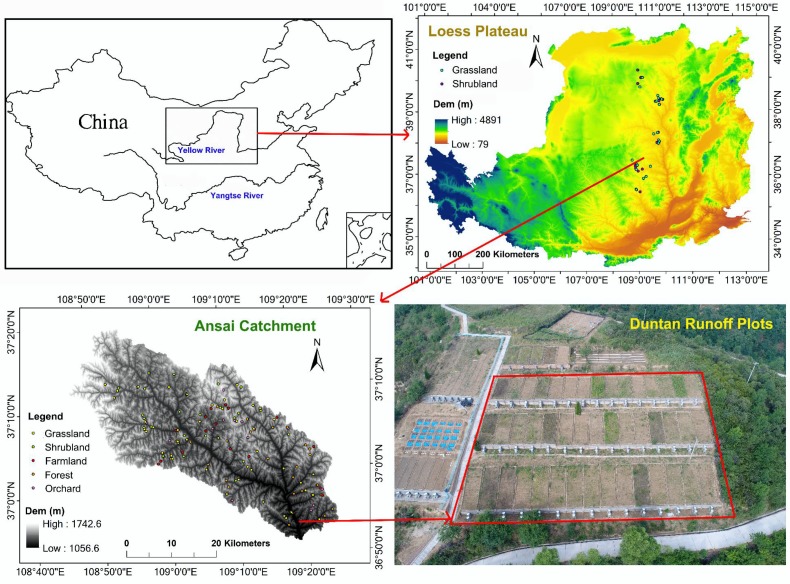
The distribution of sampling points.

The sampling plots were 2 m × 2 m for grasslands and farmland, 5 m × 5 m for shrublands and 10 m × 10 m for orchards. The distribution of the sampling points is shown in Figure 1. For the RUSLE estimate points in the runoff plots, the 0–5 cm (surface) soil samples were collected, while for the catchment and north–south sampling belt points, soil samples were collected both from the 0–5 cm layer (surface) and 5–20 cm layers (subsurface). Three repetitions were completed per sampling point, and each sample was divided into two parts after being air dried. One part was used to determine soil particle sizes using a Mastersizer 2000 (Malvern Instruments Ltd., Worcestershire, United Kingdom) after removing organic matter and dispersion with H_2_O_2_ (10%), HCl (10%) and SHMP (sodium hexametaphosphate) [92,93,94,95]. The other part was used to determine soil organic carbon with the K_2_Cr_2_O_7_ volumetric method. The soil particle size distributions were divided into sand (0.005–2.0 mm), silt (0.002–0.05 mm) and clay (<0.002 mm) with the USDA classification system [96,97]. Soil erodibility factor (*K*-factor) was chosen to represent soil erodibility of research areas [8,35,49].

### 2.3. Data Analysis

#### 2.3.1. *K* Value Based on RUSLE

The *K* value based on USLE model [8] is calculated by Equation (1):

(1) $$K=\frac{A}{R\cdot L\cdot S\cdot C\cdot P}$$

where *K* represents soil erodibility factor (short ton·ac·h/(100 ft·short ton·ac·in)); *A* represents the mean soil loss (t/(ha·year)); *R* represents the rainfall erosivity ((MJ·mm)/(ha·h)); *L* represents the slope length factor; *S* represents the slope steepness factor; *C* represents vegetation cover factor; *P* represents the support practice factor.

For Equation (1), the *R*, *L* and *S* are calculated by Equations (2)–(4) [8,91,98]:

*R* = *E* × *I*_30_ (2)

where *I*_30_ is maximum rainfall intensity over 30 min for a single rainfall and *E* represents rainfall kinetic energy [99].

*S* = 21.91 sin*θ* − 0.96 (3)

where *θ* represents the slope angle (rad).

(4) $$L=\;{\left(\frac{\lambda }{22.13}\right)}^{m}$$

where *λ* represents the length of the plot and *m* represents the slope length exponent (*m* = 0.5 if the slope is 5% or greater) [35,98].

The value of *C* and *P* in Equation (1) are set to unity with the existing studies [8,27,35,88,91].

#### 2.3.2. Estimated *K* Value

Four widely used models were chosen to calculate the *K* values: NOMO model, EPIC model, SHIRAZI model and TORRI model [99,100,101,102,103,104].

The NOMO model [47,103] is calculated as Equation (5):

*K* = [2.1×10^−4^*M*^1.14^ (12−*OM*) + 3.25 (*S*−2) + 2.5 (*P*−3)]/100 (5)

where *K* represents soil erodibility factor (short ton·ac·h/(100 ft·short ton·ac·in)); *M* represents the product of the percent of silt and very fine sand, as well as the percent of all soil fractions other than clay; *OM* represents the soil organic matter content (%); *S* represents the soil structure code; and *P* represents the soil permeability code [48,105].

The EPIC model [99] is calculated as Equation (6):

(6) $$K=\left[0.2+0.3e^{-0.0256SAN\left(1-\frac{SIL}{100}\right)}\right]{\left(\frac{SIL}{CLA+SIL}\right)}^{0.3}(1.0-\frac{0.25C}{C+e^{3.72-2.95C}})\left(1.0-\frac{0.7SN_{1}}{SN_{1}+e^{-5.51+22.9SN_{1}}}\right)$$

where *K* represents soil erodibility factor (short ton·ac·h/(100 ft·short ton·ac·in)); *SAN* represents the percent sand content; *SIL* represents the percent silt content; *CLA* represents the percent clay content; *C* represents the percent organic carbon content; and *SN1* = 1 − SAN/100.

The SHIRAZI model [104] is calculated as Equations (7) and (8):

(7) $$K=7.594\left\{0.0034+0.0405e^{-\frac{1}{2}{\left[\frac{\log\left(D_{g}\right)+1.659}{0.7101}\right]}^{2}}\right\}$$

(8) $$D_{g}\left(\mathrm{mm}\right)=e^{0.01\sum f_{i}lnm_{i}}$$

where *K* represents soil erodibility factor (short ton·ac·h/(100 ft·short ton·ac·in)); *D_g_* represents the geometric mean diameter of the soil particles; *f_i_* represents the weight percentage of the *i*-th particle size fraction (%); *m_i_* represents the arithmetic mean of the particle size limits for the *i*-th fraction (mm); and *n* represents the number of particle size fractions.

The Torri model [102] is calculated as Equations (9) and (10):

(9) $$K=0.0293\left(0.65-D_{g}+0.24{D_{g}}^{2}\right)\exp\left\{-0.0021\frac{OM}{c}-0.00037{\left(\frac{OM}{c}\right)}^{2}-4.02c+1.72c^{2}\right\}$$

(10) $$D_{g}=\sum f_{i}lg\sqrt{d_{i}d_{i-1}}$$

where *K* represents soil erodibility factor (short ton·ac·h/(100 ft·short ton·ac·in)); *OM* represents the percent content of soil organic matter; *c* represents the percent content of clay; *D_g_* represents the Napierian logarithm of the geometric mean of the particle size distribution; *d_i_* (mm) represents the maximum diameter of the *i*-th class; *d_i−1_* (mm) represents the minimum diameter; *f_i_* represents the mass fraction of the corresponding particle size class.

To compare among different models, all the results in the United States customary units were multiplied by 0.1317 to convert to the international units for the *K* values (t·hm^2^·h/(MJ·mm·hm^2^)).

The one-way analysis of variance (ANOVA) and the least squares difference method (LSD) were used to analyze and verify the values among different models and places. Data pretreatment was performed using Excel 2016. One-way ANOVA and correlation and significance tests were conducted using SPSS 23.0 (IBM, Armonk, NY, USA). The figures were plotted using ArcGIS 10.0 (Redlands, CA, USA), Origin 9.2 (Origin Lab Corporation, Northampton, MA, USA) and R Studio 1.1.447 (RStudio Inc., Boston, MA, USA).

## 3. Results

### 3.1. The K Value in RUSLE Estimate Points

The *K* values calculated by RUSLE estimate data from runoff plots are shown in Table 1. The *K* value varies with slope and land uses. For the 5° slope, the *K* value ranges from 0.005 to 0.019 (t·hm^2^·h/(MJ·mm·hm^2^)). The *K* values decrease in shrubland and increase in natural grassland with the increase of slope, while the values differ in introduced grassland and farmland. For each slope, farmland has a higher *K* value than plots with other land uses.

The *K* values estimated by NOMO model, EPIC model, SHIRAZI model and TORRI model are shown in Table 2. The *K* value ranges from 0.089 to 0.100 with NOMO model, from 0.045 to 0.050 with EPIC model, from 0.047 to 0.049 with SHIRAZI model and from 0.013 to 0.060 with TORRI model (unit: t·hm^2^·h/(MJ·mm·hm^2^)). The standard deviation (SD) of *K* values with each model are *K*-NOMO (0.003), *K*-EPIC (0.002), *K*-SHIRAZI (0.001) and *K*-TORRI (0.012). The coefficient of variation (CV) of *K* values with each model are *K*-NOMO (0.031), *K*-EPIC (0.033), *K*-SHIRAZI (0.012) and *K*-TORRI (0.253).

**Figure 2:**
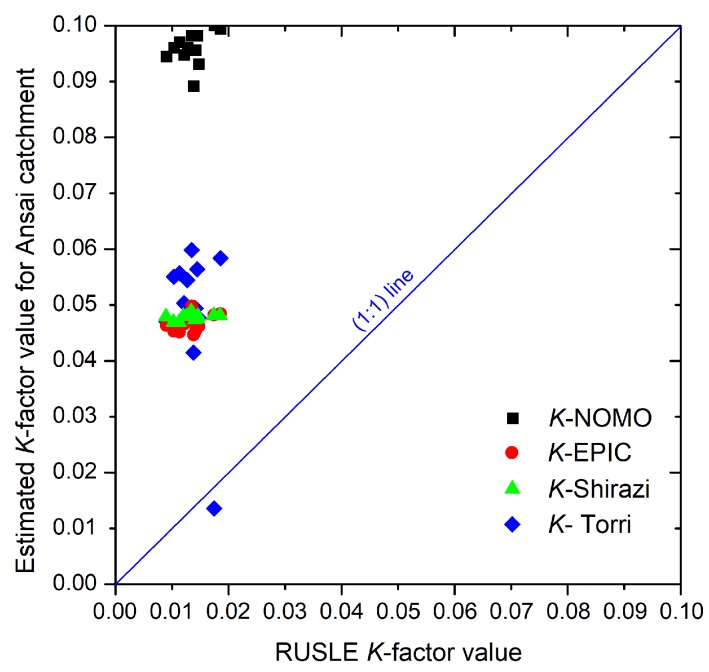
The comparison of the RUSLE and other estimated *K* values for 12 RUSLE soils at catchment scale (unit: t·hm^2^·h/(MJ·mm·hm^2^)).

The comparison of *K* values between RUSLE *K*-factor values and other estimated values are shown in Figure 2. Most of the data are located under the 1:1 line. The different models show different relationships to RUSLE values.

### 3.2. The Comparison between the Modeling Results of K Value and the RUSLE Estimate Value

**Figure 3:**
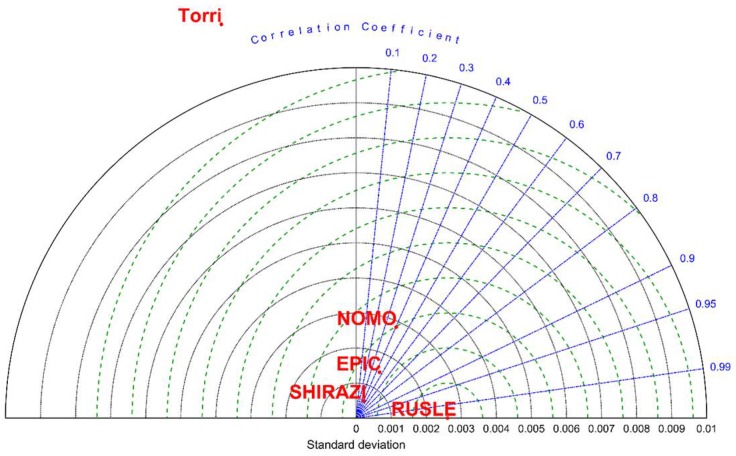
The Taylor analysis of different models.

The comparison of different models is shown in Figure 3. The root-mean-square error (RMSE), standard deviation (SD) and correlation coefficient are all shown in the Taylor diagram [106]. The TORRI model shows obvious difference from other models. The SD of NOMO model is closer to the RUSLE estimate values than other models, while the correlation coefficients of SHIRAZI model and EPIC model are closer to 1 than other models. The differences of SD and RMSE between EPIC model and SHIRAZI model are not obvious. When taking SD, RMSE, correlation coefficient and number of parameters of each model into consideration, SHIRAZI model was chosen to apply at catchment scale and regional scale.

### 3.3. The Distribution of Shrubland and Grassland K Values at Catchment and Regional Scales

**Figure 4:**
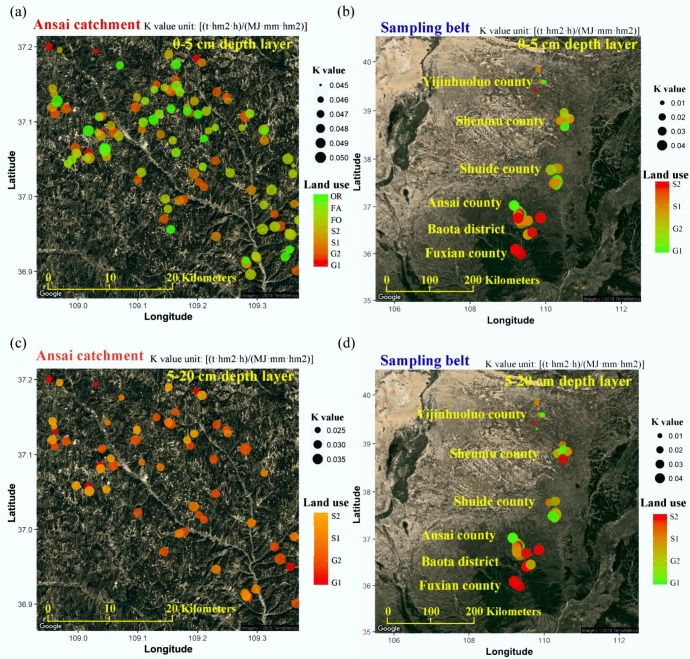
The distribution of *K* values at catchment ((**a**) for 0–5 cm depth layer and (**c**) for 5–20 cm depth layer) and region scale ((**b**) for 0–5 cm depth layer and (**d**) for 5–20 cm depth layer) by using the SHIRAZI model. G1 represents introduced grassland; G2 represents natural grassland; S1 represents introduced shrubland; S2 represents natural shrubland; FO represents forest; FA represents farmland; OR represents orchard; the different colors of bubbles represent different dominant species; the size of the bubbles represents the *K* value.

The *K* values of shrubland and grassland at catchment and regional scales were calculated by SHIRAZI model, and the results are shown in Figure 4, Table 3 and Table 4.

At catchment scale (Figure 4 and Table 3), the mean *K* values of introduced grassland are clearly lower than other land use types in both 0–5 cm and 5–20 cm soil layers. For the grassland and shrubland, the *K* values of the 5–20 cm soil layer range from 0.025 to 0.035 (t·hm^2^·h/(MJ·mm·hm^2^)) and the mean *K* values are lower than that in the 0–5 cm soil layers.

At regional scale (Figure 4 and Table 4), the mean *K* values increase from north to south along the sampling belt. The *K* values of G1, G2, S1 and S2 in the 0–5 cm layer vary from 0.008 to 0.037, 0.031 to 0.046, 0.012 to 0.041 and 0.008 to 0.045 (t·hm^2^·h/(MJ·mm·hm^2^)). The *K* values of G1, G2, S1 and S2 in the 5–20 cm layer vary from 0.009 to 0.039, 0.032 to 0.046, 0.012 to 0.042 and 0.008 to 0.048 (t·hm^2^·h/(MJ·mm·hm^2^)). From Shenmu county to Suide county, most of the *K* values of introduced vegetation (G1 and S1) are lower than those of natural vegetation (G2 and S2). The differences between introduced and natural vegetation are not obvious in Baota county and Fuxian county. For G1, G2 and S2, most of the *K* values in the 0–5 cm layers are lower than those in the 5–20 cm layers.

## 4. Discussion

### 4.1. Comparison of the Soil Erodibility Models Used in Loess Plateau

According to the results from this paper and existing studies, the estimated *K* values change in different models and have clear differences with the RUSLE *K*-factor value [8,34,35,45]. Even for the same species in the same slope, the maximum value of estimate *K* value could be four times the minimum value (Table 2, Figure 3). All the estimated *K* values in this paper are higher than the RUSLE estimate *K* values (Table 2, Figure 3). It is difficult to measure *K* value in the field because it needs a long observation time and the runoff plots must be in good condition [8]. Existing studies have shown that the *K* value is influenced by many factors, and the factors are always changing [4,8]. In addition, different kinds of erosion may come together when erosion occurs, and the intensity of each kind of erosion is influenced by many factors as well [8]. So, it is also difficult to estimate *K* value accurately when only using a few kinds of soil properties. The *K* values which are calculated by USLE/RUSLE model are usually regarded as the benchmark value, although the complex index in USLE/RUSLE increases the uncertainty of the results [91,107].

Among the four models mentioned in this paper, the NOMO model considers the soil particle size distribution, soil organic matter and soil structure characteristics [101]; the EPIC model and TORRI model consider the soil particle size distribution and soil organic carbon [100,102]; while the SHIRAZI model only considers the soil particle size distribution [104]. There are many ways to evaluate a model. RMSE, SD and correlation coefficient were used to evaluate models in this study. As the existing studies showed, the estimate values are more credible when the RMSE becomes closer to 0, the SD becomes closer to the SD of RUSLE *K*-factor value and the correlation coefficient becomes closer to 1 (Figure 3, Table 2). In this study, the *K*-TORRI values show obvious differences from the RUSLE *K*-factor value and other models, so this model was excluded first (Figure 3). The NOMO model is suitable for the middle-western part of America with low aggregate structure and medium-textured soils. The NOMO model performs better in terms of SD than other models in our research area, while the RMSE and correlation coefficient are similar in EPIC and SHIRAZI models (Figure 3). The NOMO model also needs more parameters than other models, which makes it hard to use at a large scale, because the large scale usually lacks detailed data. So, EPIC and SHIRAZI models are more suitable in our research areas. As SHIRAZI model needs fewer parameters than EPIC model, SHIRAZI model was chosen to estimate *K* values at catchment and regional scales in this study.

### 4.2. K Value Variation of Different Kinds of Land Use Types at Catchment Scales

The *K* values of catchment scales show obvious differences between 0–5 cm soil layers and 5–20 cm soil layers (Table 3, Figure 4). For the 0–5 cm layers, this study takes seven kinds of land use types into consideration. According to existing studies, soil erosion of farmland is the most serious [37]. In this study, *K* values in farmland are similar to other kinds of vegetation (Table 3). It indicates that the *Grain to Green* project has remarkable results. This project returns farmland into forest, shrub or grass in the areas which are easily eroded, and it also encourages farmers to build terraces on slopes. Both of these practices can protect soil from erosion. In the same small watershed in the catchment, surface *K* values change with the position and the dominant species of the sampling points. However, at the catchment scale, mean 0–5 cm *K* values of most kinds of vegetation are similar to each other. Only the *K* values of G1 are obviously lower than others, which indicates the introduced grasslands (usually dominated with leguminous plants) are better for soil conservation, while previous studies showed that the introduced grasslands use more water and may cause unrecoverable loss of soil moisture in deep soil layers (usually in 500–800 cm depth) in the short term [108,109,110]. For the Ansai catchment, the planting area and age of introduced grassland should be controlled to protect deep soil moisture, although it has a good performance in protecting surface soil particles.

For the 5–20 cm soil layers, only the most widely distributed land use types (G1, G2, S1, S2) in this catchment were analyzed (Table 3, Figure 4). The *K* values of 5–20 cm soil layers show the magnitude of potential erosion risk. The 5–20 cm *K* values of these four kinds of land use types are all lower than the 0–5 cm values, while the variations show differences among vegetation covers (Table 3). The *K* values in introduced shrublands and in natural shrublands are similar to each other in this layer. The *K* values of introduced grassland are lower than those in shrublands, which are similar to the 0–5 cm layer, while the *K* values of natural grassland (usually dominated with Gramineae plants) are higher than those in shrublands in 5–20 cm soil layers. It may be because the roots of most Gramineae plants are weak and their leaves are soft [110,111,112,113]. In that case, the leaves can hardly slow down the drops of rainfall or prevent soil from being detached by runoff. Although soil moisture in natural grassland is higher than in other land use types of this catchment, natural grassland is not good for soil protection.

### 4.3. K Value Variation of Shrubland and Grassland at Regional Scale

Different from the catchment scale, the *K* values do not show obvious differences between 0–5 cm layers and 5–20 cm layers at regional scale (Table 4, Figure 4). With the expansion of the study area, the differences in environmental factors and soil factors of sampling points gradually increase, and the differences in *K* value increase as well. Previously studies have shown that the amount of precipitation and the percentage of clay in soil increase from north to the south along the sampling belt [81]. In the sampling areas, nearly 20 years have passed since the beginning of *Grain to Green* project, and the vegetation coverage increases continuously [114,115,116]. In this study, the *K* values show obvious differences in different sampling areas (Table 4, Figure 4). The Yijinhuoluo county has a multiyear precipitation of 310–370 mm [84], and the *K* values are the lowest in this sampling belt (Table 4, Figure 4). The Shenmu county, Suide county and the north part of the Ansai county have a multiyear precipitation of 370–440 mm [84]. In these two areas, the *K* values of introduced vegetation are lower than that of natural vegetation. The *K* values of introduced grassland are higher than those of introduced shrubland, while the *K* values of natural grassland are lower than those of natural shrubland. The Baota district and the south part of the Ansai county have multiyear precipitations of 440–510 mm [84]. In these areas, the *K* values of introduced vegetation are higher than those of natural vegetation, which shows different trends with the 370–440 mm areas (Table 4). The Fuxian county has a multiyear precipitation of 510–580 mm [84]. In this area, the soil moisture is high enough to support most of the plants’ growth, so there is little introduced vegetation here. For the natural vegetation, the *K* values of grassland are higher than those of shrubland in 0–5 cm layers, while the *K* values of grassland are lower than those of shrubland in 5–20 cm layers (Table 4).

According to previous studies, the areas with a multiyear precipitation of 370–440 mm are those where the vegetation has the greatest impact on and response from soil moisture [84]. In this study, these areas are also the places where differences in *K* values among land uses are larger than other areas (Figure 4). In addition, these areas also belong to the hilly region of the Loess Plateau, where the erosion is heavier than in other places [46]. In the north part of the sampling belt, the lower soil moisture limits the growth of plants, so all kinds of plants have low vitality; in the south part of the sampling belt, the higher soil moisture can support the growth of nearly all kinds of plants, so the plants can grow healthily and protect the soil better. In that case, the vegetation restoration in the middle part of the sampling belt needs to concentrate on the species, plant area and plant age.

## 5. Conclusions

This study compares the four commonly used *K* value estimate models, including NOMO model, EPIC model, SHIRAZI model and TORRI model. Both the EPIC model and SHIRAZI model are suitable to estimate the *K* value in the Loess Plateau. The SHIRAZI model has the advantage of fewer parameters, so it is more suitable for a large scale, where the data are usually incomplete. For the catchment scale, introduced grass has better ability to protect both the 0–5 cm soils and 5–20 cm soils, while the differences between introduced and natural shrubland are not obvious. For regional scale, the differences between the 0–5 cm layers and 5–20 cm layers are not obvious in all the introduced and natural shrubland and grassland. The *K* values increase with the increase of mean multiyear precipitation, and the areas with a mean multiyear precipitation of 370–440 mm are the most important places for vegetation restoration construction management. The introduced grass and shrub show better ability in protecting soils, though they use too much soil moisture after a long period of growth. The soil moisture in natural grassland is higher than in other land use types of most research areas, but these kinds of plants are not good for soil protection. In that case, a comprehensive balance between water conservation and soil conservation is needed and important when selecting the species used for vegetation restoration in arid and semiarid areas.

## Figures and Tables

**Table 1:** The mean value of *K* in runoff plots (revised universal soil loss equation (RUSLE) estimate (t·hm^2^·h/(MJ·mm·hm^2^)).

Slope	Introduced Grass (G1)	Natural Grass (G2)	Farmland (FA)	Natural Shrubland (S2)
5°	0.011	0.009	0.019	0.014
15°	0.014	0.013	0.014	0.008
25°	0.010	0.014	0.017	0.005

**Table 2:** The estimated value of *K* used to compare models at Ansai catchment (t·hm^2^·h/(MJ·mm·hm^2^)).

Species	Slope	Number of Samples	*K*-NOMO	*K*-EPIC	*K*-SHIRAZI	*K*-TORRI
Introduced grass (G1)	<10°	4	0.097	0.045	0.047	0.056
	10°–20°	4	0.096	0.045	0.047	0.049
	>20°	3	0.096	0.045	0.047	0.055
Natural grass (G2)	<10°	7	0.094	0.046	0.048	0.047
	10°–20°	8	0.096	0.047	0.048	0.054
	>20°	9	0.098	0.050	0.049	0.060
Shrubland (S2)	<10°	6	0.089	0.045	0.048	0.041
	10°–20°	14	0.095	0.046	0.048	0.050
	>20°	7	0.093	0.046	0.048	0.048
Farmland (FA)	<10°	13	0.099	0.048	0.048	0.058
	10°–20°	3	0.098	0.046	0.048	0.056
	>20°	1	0.100	0.048	0.048	0.013

**Table 3:** The mean value of *K* at catchment scale (t·hm^2^·h/(MJ·mm·hm^2^)).

Depth	G1	G2	S1	S2	FO	FA	OR
0–5 cm	0.047	0.048	0.048	0.048	0.048	0.048	0.048
5–20 cm	0.027	0.032	0.030	0.030	-	-	-

**Note:** G1 represents introduced grassland; G2 represents natural grassland; S1 represents introduced shrubland; S2 represents natural shrubland; FO represents forest; FA represents farmland; OR represents orchard.

**Table 4:** The mean value of *K* at regional scale (t·hm^2^·h/(MJ·mm·hm^2^)).

County	0–5 cm				5–20 cm			
	G1	G2	S1	S2	G1	G2	S1	S2
Yijinhuoluo	0.008	-	0.012	0.008	0.009	-	0.012	0.008
Shenmu	0.028	0.031	0.021	0.032	0.030	0.032	0.021	0.032
Suide	0.036	0.038	0.036	0.040	0.037	0.038	0.032	0.040
Ansai	0.037	0.036	0.035	0.031	0.039	0.037	0.035	0.032
Baota	-	0.040	0.041	0.041	-	0.040	0.042	0.042
Fuxian	-	0.046	-	0.045	-	0.046	-	0.048

**Note:** G1 represents introduced grassland; G2 represents natural grassland; S1 represents introduced shrubland; S2 represents natural shrubland.
